# High Spatiotemporal Resolution Mapping of Surface Water in the Southwest Poyang Lake and Its Responses to Climate Oscillations

**DOI:** 10.3390/s20174872

**Published:** 2020-08-28

**Authors:** Haifeng Tian, Jian Wang, Jie Pei, Yaochen Qin, Lijun Zhang, Yongjiu Wang

**Affiliations:** 1 Key Laboratory of Geospatial Technology for the Middle and Lower Yellow River Regions of Ministry of Education/College of Environment and Planning, Henan University, Kaifeng 475001, China; zlj7happy@vip.henu.edu.cn (L.Z.); 1813060007@vip.henu.edu.cn (Y.W.); 2 Department of Geography, The Ohio State University, Columbus, OH 43210, USA; wang.12679@osu.edu; 3 School of Geospatial Engineering and Science, Sun Yat-Sen University, Zhuhai 519000, China; peij5@mail.sysu.edu.cn

**Keywords:** Poyang Lake, surface water, Sentinel-1, Google Earth Engine, remote sensing, SWI

## Abstract

Accurately quantifying spatiotemporal changes in surface water is essential for water resources management, nevertheless, the dynamics of Poyang Lake surface water areas with high spatiotemporal resolution, as well as its responses to climate change, still face considerable uncertainties. Using the time series of Sentinel-1 images with 6- or 12-day intervals, the Sentinel-1 water index (SWI), and SWI-based water extraction model (SWIM) from 2015 to 2020 were used to document and study the short-term characteristics of southwest Poyang Lake surface water. The results showed that the overall accuracy of surface water area was satisfactory with an average of 91.92%, and the surface water area ranged from 129.06 km^2^ on 2 March 2017 to 1042.57 km^2^ on 17 July 2016, with significant intra- and inter-month variability. Within the 6-day interval, the maximum change of lake area was 233.42 km^2^ (i.e., increasing from 474.70 km^2^ up to 708.12 km^2^). We found that the correlation coefficient between the water area and the 45-day accumulated precipitation reached to 0.75 (*p* < 0.001). Moreover, a prediction model was built to predict the water area based on climate records. These results highlight the significance of high spatiotemporal resolution mapping for surface water in the erratic southwest Poyang Lake under a changing climate. The automated water extraction algorithm proposed in this study has potential applications in delineating surface water dynamics at broad geographic scales.

## 1. Introduction

Lakes are among the most important terrestrial water resources. Information regarding lake surface waters is fundamental for supporting ecosystem services [1], especially wetlands [2] and biodiversity assessments [3], serving as a critical indicator to assess changes in local ecosystems at nearby lakes [4]. Besides, flooding is a natural disaster that causes the most economic loss and casualties. Timely observations of the extent of floodwater are needed for rapid disaster response and management [5]. There are some existing water map products used to depict the extent of global water, such as the Joint Research Centre of the European Commission (JRC) global surface water map [6], global open permanent water bodies [7] and global raster water mask at 250 m resolution [8]. However, these products cannot quickly characterize variations in the surface water due to a lack of sufficient spatial and/or temporal resolutions [9].

Poyang Lake is well known for its ecological and economic importance, as well as rapid changes in lake inundation areas [10,11,12,13]. Optical remote sensing images enable high accuracy surface water identification [14,15,16]. Some spectral water indexes based on such imagery include the normalized difference water index (NDWI) [17], the modified normalized difference water index (MNDWI) [14], and the automated water extraction index (AWEI) [18]. However, two challenges remain using optical imagery to measure high spatiotemporal changes in the surface water of southwest Poyang Lake, which is the study area in this paper.

First, southwest Poyang Lake is located in a subtropical area. Rainy weather in the region results in limited high-quality cloud-free optical images, such as from the non-commercial Landsat or Sentinel-2 platforms, to detect detailed inundation changes. For example, our previous research showed that there were only eight available Landsat-8 images spanning from 24 May 2015 to 14 November 2016 covering Poyang Lake; yet, another 27 Landsat-8 images were heavily contaminated by clouds [4].

Second, southwest Poyang Lake is composed of numerous isolated lakes and small rivers, especially during the dry seasons. Coarse spatial resolution images, e.g., Moderate Resolution Imaging Spectroradiometer (MODIS) images with a spatial resolution of 250/1000 m, provide insufficiently satisfactory classification accuracy [4,19,20], due to the mixed water and non-water pixels. Furthermore, there are only one or two cloudless MODIS images in many calendar months within a year, even if the MODIS images have a daily temporal resolution [10,21]. Consequently, optical remote sensing satellites, with a lack of available high-quality data have strong limitations in their ability to monitor rapid changes in the surface water extent of southwest Poyang Lake. 

Synthetic aperture radar (SAR) is particularly suitable for flood mapping as they provide full-day observations regardless of adverse illumination or weather conditions [22,23,24]. Sentinel-1 has a 6 to 12 day repeat, depending on the area, and has an open access mechanism starting from 2015. There is mounting evidence that Sentinel-1 can be used to identify water bodies and quantify surface water extents [9,22,25,26]. For example, Zeng et al. [19] used the threshold value of -16.35 dB and the Sentinel-1 VV-polarization imagery to extract the Poyang Lake water extent without evaluating the classification accuracy. Hu et al. [9] proposed a heuristic and automated water extraction method to extract the water map from the Sentinel-1 data with an overall accuracy between 94.32% and 96.7%. Vickers, et al. [27] used the unsupervised K-means clustering algorithm along with the Sentinel-1 and RADARSAT-2 imagery to create binary maps of the water area. Besides, some data-driven methods using machine learning algorithms were widely used in previous studies [28]. For example, Huang et al. [29] proposed a fully automatic classification tree approach to classify surface water extent using Sentinel-1 data with an overall accuracy of 82% to 93%.

Among these previous methods to identify the surface water extent based on Sentinel-1 imagery, threshold-based methods have been the most widely used, in part because they are computationally less expensive and can yield accuracies comparable to more complex segmentation approaches [22]. The thresholds are generally determined by analyzing histograms of the SAR backscatter intensity and then estimating the probability distributions of water and non-water pixels. Tian et al. [4] proposed the Sentinel-1 Water Index (SWI) based on a linear combination of the VV- and VH- polarization images and illustrated that SWI could more reliably identify water bodies than the traditional threshold method that directly uses VV- and/or VH- polarization imagery. Therefore, we adopted the SWI threshold-based method and mapped the surface water in southwest Poyang Lake using the Google Earth Engine (GEE) cloud computing platform, which has the advantage of rapidly processing remote sensing big data and has been widely recognized and applied in academia [30,31,32,33,34,35,36].

Although drastic fluctuations in the surface water area are the result of comprehensive influences from multiple factors, some scholars believe that atmospheric precipitation is the dominant factor that drives changes in the surface water area of Poyang Lake. For example, Zeng et al. [19] noted that the correlation coefficient between the surface water area of Poyang Lake and the precipitation data was 0.64 (*p* < 0.05) at the month scale. It is well known that there is a lag effect of precipitation on the lake water content due to the process of surface runoff merging into the lake. Few works have considered what number of days of accumulated precipitation is most correlated with the surface water for the southwest Poyang Lake.

Therefore, our contribution to the study of the southwest Poyang Lake surface water dynamics is as follows. (1) A time-series surface water dataset for the southwest Poyang Lake with a 6- or 12-day interval at a 30-m spatial resolution spanning from May 2015 to June 2020 was produced for the first time. (2) The correlation between the southwest Poyang Lake surface water area and the accumulated precipitation over different days was analyzed to reveal the impact of climate oscillations on changes in water areas.

## 2. Methodology and Materials

### 2.1. Study Area

**Figure 1:**
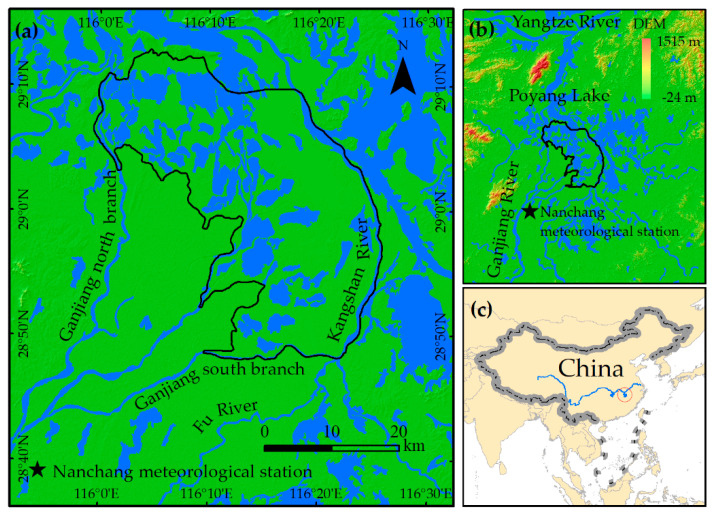
Location and distribution map of the study area. (**a**) The boundary and surroundings of the study area where the blue region is the surface water derived from the Sentinel-1 imagery on 3 May 2020 and the pentagram symbol indicates the location of the meteorological station. (**b**) The location of the study area in the Poyang Lake and (**c**) the location of Poyang Lake in China.

The southwest of Poyang Lake was selected as the study area, as shown in Figure 1, as the water dynamics here are more volatile. The study area was divided into many isolated sub-lakes during the dry seasons. The eastern and southern boundaries of the study area are the Kangshan River and the south branch of the Ganjiang River, respectively. The western boundary is along the north branch of the Ganjiang River and the west bank of Poyang Lake, and the northern boundary is the mid-lake island. The total area of the study region is approximately 1059 km^2^, which is around one-third of the total area of Poyang Lake.

The southwest of Poyang Lake is an important part of the Poyang Lake wetland [37]. The climate type of the study area belongs to subtropical monsoon climate. It is the rainy season from April to September and the dry season from October to March. The annual total precipitation is approximately 1600 mm, and the annual average temperature is 25 °C [38].

### 2.2. Datasets and Preprocessing

#### 2.2.1. Sentinel-1 Imagery

Sentinel-1 is a two-satellite polar-orbiting constellation (Sentinel-1 A and B) governed by the European Space Agency (ESA) [39]. The revisit time for a single satellite is 12 days [40], and the two-satellite constellation could provide a revisit time of 6 days in some regions [41]. The Sentinel-1 mission provides dual-polarization data (VH and VV) in the C-band (center frequency of 5.405 GHz) as a SAR instrument [42]. The Sentinel-1 data collection stored in the GEE is the “COPERNICUS/S1_GRD”. This collection includes the Sentinel-1 ground range detected (GRD) scenes as processed using the Sentinel-1 Toolbox to generate a calibrated, ortho-corrected product, which backscattering coefficient is less than 0 dB. These Sentinel-1 images used in the study have a wide coverage of 250 km with a spatial interval of 10 m. To reduce the effects of coherent speckle noise, a median filter was used with a window size of 3 pixels by 3 pixels. Besides, the spatial resolution of the Sentinel-1 imagery was downsampled to 30 m by 30 m in this study. In this study, there were a total of 255 images from 24 May 2015 to 2 June 2020 acquired to identify surface water for the southwest Poyang Lake. From 24 May 2015 to 15 September 2016, there were only 36 Sentinel-1A images with a 12-day interval, excluding five missing images. From 27 September 2016 to 2 June 2020, there were 219 Sentinel-1A/B images with a 6-day interval, excluding six missing images.

#### 2.2.2. Landsat-8 Imagery

The Landsat-8 data collection stored in the GEE cloud platform is “LANDSAT/LC08/C01/T1_SR”, which is the atmospherically corrected surface reflectance from the Landsat-8 OLI/TIRS sensors. These images are composed of five visible and near-infrared bands and two short-wave infrared bands processed to the orthorectified surface reflectance and two thermal infrared bands processed to the orthorectified brightness temperature with a spatial resolution of 30 m. In this study, Landsat-8 imagery was used to build accuracy validation samples because Landsat-8 image demonstrates good performance in identifying water surfaces [6,43,44]. A total of six Landsat-8 images without cloud cover were used during the study period, and their imaging dates were the same as those of the Sentinel images from 9 September 2015, 27 September 2016, 25 May 2017, 2 July 2019, 19 August 2019, and 15 April 2020. The modified normalized difference water index (MNDWI) is an effective way to extract water bodies [14,45,46]. In this study, the MNDWI image was obtained from [14]:

(1) $$MNDWI=\left(\beta_{green}-\beta_{SWIR}\right)/\left(\beta_{green}+\beta_{SWIR}\right)$$

where *β_green_* and *β_SWIR_* represent the surface reflectance of the green band (B3 of Landsat-8 imagery) and the first shortwave infrared (SWIR) band (B6 of Landsat-8 imagery), respectively. Moreover, the normalized difference vegetation index (NDVI) image was derived from the Landsat-8 image as [47,48]:

(2) $$NDVI=\left(\beta_{NIR}-\beta_{red}\right)/\left(\beta_{NIR}+\beta_{red}\right)$$

where *β_NIR_* and *β_red_* represent the surface reflectance of the near-infrared (NIR) band (B5 of Landsat-8 imagery) and the red band (B4 of Landsat-8 imagery), respectively. We then used the unsupervised k-means classification integrated into the ENVI software to identify the surface water of southwest Poyang Lake based on the MNDWI and NDVI images.

#### 2.2.3. Meteorological Data

Previous research considered precipitation as one of the most dominant factors that dictate changes in the Poyang Lake surface water [10,19]. Therefore, we considered the correlation between the southwest Poyang Lake surface water area and the daily precipitation data from meteorological station using the Pearson correlation analysis [49,50]. The daily precipitation data from Nanchang meteorological station (115.55 E, 28.36 N, Figure 1a was used, which were obtained from the China National Meteorological Science Data Center at http://data.cma.cn/data/cdcindex.html. Nanchang meteorological station is located upstream of the study area where the closest distance to the area is 30 km. Therefore, we believe that it is appropriate to conduct a correlation analysis between the precipitation data from this station and the surface water area. As the influence of precipitation on hydrology is time-lagged, we analyzed the relationships between the 1–80-day accumulated precipitation and the southwest Poyang Lake surface water area to explore how many days of accumulated precipitation are most correlated with the southwest Poyang Lake surface water area. For example, the 10-day accumulated precipitation for the 15 January 2020 means the total amount of precipitation among 10 days before this day, i.e., from 5 January 2020 to 15 January 2020.

There are three daily precipitation record times from the Nanchang meteorological station. The first is from 8 p.m. the previous day to 8 a.m. that day. The second is from 8 a.m. to 8 p.m. the same day. The third is from 8 p.m. the previous day to 8 p.m. that day. The imaging time of the Sentinel-1 satellite is approximately 10 a.m. local time and the precipitation after imaging will not affect the water distribution derived from the image. Therefore, we used the first type of daily precipitation record to represent the daily precipitation for the imaging day of the Sentinel-1 images. We used the third type of precipitation record to represent the daily precipitation for other days. For example, for the surface water area on 24 May 2015, the 1-day accumulated precipitation is from 8 p.m. 23 May 2015 to 8 a.m. 24 May 2015, the 2-day accumulated precipitation is from 8 p.m. 22 May 2015 to 8 a.m. 24 May 2015, and the 3-day accumulated precipitation is from 8 p.m. 21 May 2015 to 8 a.m. 24 May 2015. Therefore, the accumulated precipitation data of the 1–80-day segments were obtained, which were then correlated with the surface water area on 24 May 2015.

### 2.3. Sentinel-1 Water Extraction Model

Our previous study [4] proposed a simple but robust SWI-based water extraction model (SWIM) derived from Sentinel-1 imagery to extract the spatial distribution of water areas. The SWI was computed as:

(3) $$SWI=0.1747\times \beta_{vv}+0.0082\times \beta_{vh}\times \beta_{vv}+0.0023\times \beta_{vv}{}^{2}-0.0015\times \beta_{vh}{}^{2}+0.1904$$

where *β_vh_* and *β_vv_* represent the backscattering coefficients in for VH and VV polarization, respectively. Based on our previous results, if the SWI value of one pixel is more than 0.2, it is regarded as a water body [4]. The specific codes of SWIM on GEE are as shown in Appendix A.

### 2.4. Accuracy Validation

The water extent extracted from the Landsat-8 imagery was regarded as the ground truth to examine the classification accuracy as referenced in previous research [4,43]. Hence, in our study, the surface water derived from Landsat-8 images was selected as the reference data to validate the classification accuracy using the confusion matrix method [51,52]. The main parameters of confusion matrix accuracy validation are the overall accuracy, the user’s accuracy, the producer’s accuracy, and the kappa coefficient. The user’s accuracy is complementary to the commission, and the producer’s accuracy is complementary to omission. For specific methods, please refer to the literature [51,52].

## 3. Results

### 3.1. Accuracy 

**Figure 2:**
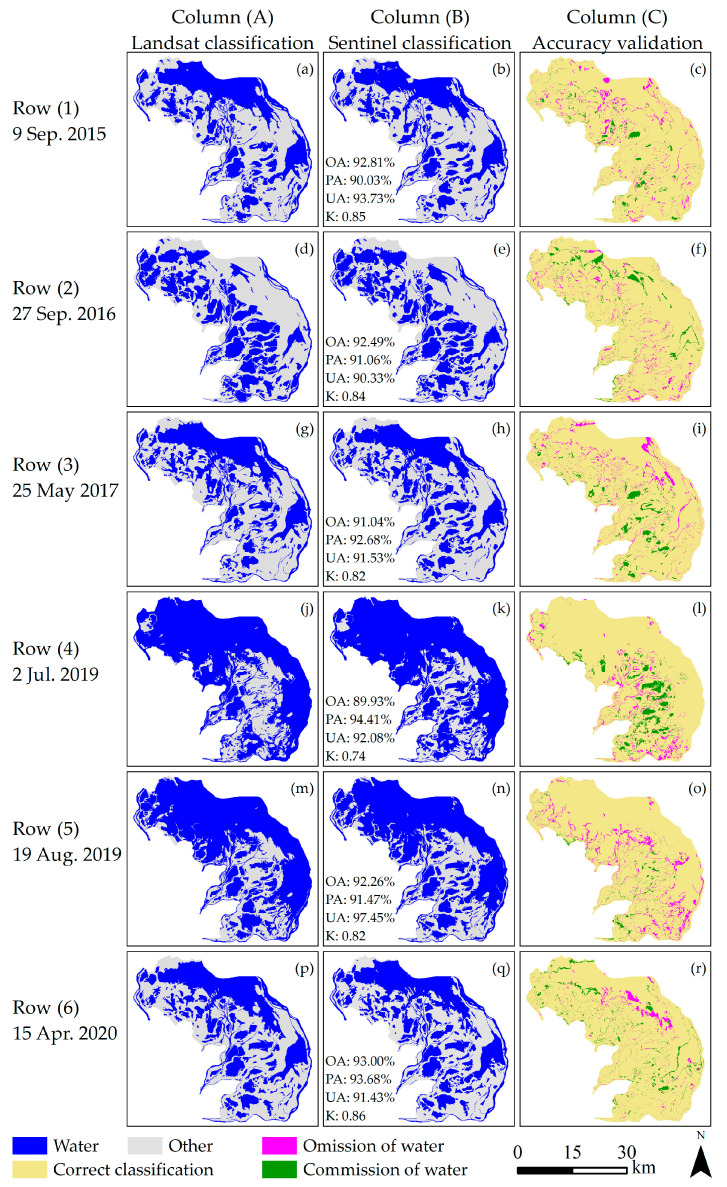
Accuracy verification for the Sentinel-1 image-derived classification compared with the Landsat classification results. OA = overall accuracy, UA = user’s accuracy for water classification, PA = producer’s accuracy for water classification, and K = kappa coefficient.

For the accuracy validation results over the six periods, as shown in Figure 2, the average overall accuracy is 91.92%, with an average Kappa coefficient of 0.82. The user’s and producer’s accuracies are very similar for each evaluation result with average values of 92.76% and 92.22%, respectively. The period with the overall accuracy (89.93%) is 2 July 2019, which is because there are many commission error according to Figure 2l. The highest overall accuracy was 93.00% on 145 April 2020. Most errors are distributed at the water-land boundary, although there exist some large error patches with an area of approximately 4 km^2^.

### 3.2. Inundation Dynamics

**Figure 3:**
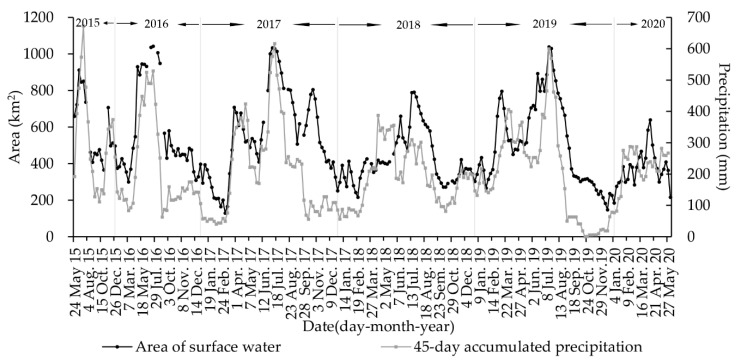
Time-series for changes in the surface water area of southwest Poyang Lake from 24 May 2015 to 2 June 2020. There are some data gaps in the area curve due to Sentinel-1 imagery unavailability during some time points. The period from 24 May 2015 to 15 September 2016 has a 12-day interval, and the period from 27 September 2016 to 2 June 2020 has a 6-day interval. The dotted line is the accumulated precipitation within 45 days prior to the corresponding date, which shows a high correlation with the surface water area.

The time series of surface water area for southwest Poyang Lake from 24 May 2015 to 2 June 2020 is plotted in Figure 3 which illustrated the area varied dramatically at both month and year scales. During the study period, the maximum area of surface water was 1042.57 km^2^ on 17 July 2016, which is seven times larger than the minimum area of 129.06 km^2^ on 2 March 2017. The average area was 500.61 km^2^ with a standard deviation of 214.38 km^2^.

Within 6 days, the maximum changed area was 233.42 km^2^ from 474.70 km^2^ on 20 March 2017 up to 708.12 km^2^ on 26 March 2017, and the change rate reached 49.17%. The average changed area was 55.81 km^2^ with a change rate of 13.21%. Water surface changes within 12 days were more clustered. For example, the maximum changed area was 382.88 km^2^ from 547.63 km^2^ on 12 April 2016 up to 930.51 km^2^ on 24 April 2016, and the change rate reached 69.92%.

There were multiple peaks (1 to 3) for the temporal dynamics of the surface water area within a year with regular occurrence (i.e., once) in July for each year. In addition, there was significant differences in the peak values and durations. For instance, the maximum surface water area in 2018 was less than 800 km^2^. In contrast, the maximum surface water area in 2016 was more than 1000 km^2^. Besides, the number of days with surface water area of more than 800 km^2^ was at least 160 days in 2016. As a result, the average annual area was 568.62 km^2^, 549.06 km^2^, 428.24 km^2^, and 514.08 km^2^ in 2016, 2017, 2018, and 2019, respectively.

**Figure 4:**
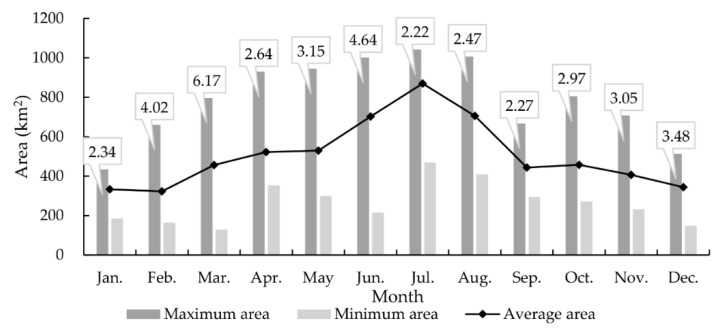
The maximum, minimum, and average areas of the southwest Poyang Lake surface water for each month calculated based on multi-year observations. The marked number is the ratio of the maximum to the minimum area.

The monthly analysis of the surface water area of southwest Poyang Lake as calculated from the average of multi-year observations for each calendar month is illustrated in Figure 4. Changes in the surface water area within a calendar month are rather dramatic. For example, the average ratio of the maximum to minimum areas within a calendar month was 3.29. The most drastic fluctuations in the surface water area occurred during March with a maximum/minimum ratio of 6.17. Additionally, for the monthly average area of the surface water, the maximum and minimum monthly averages were 869.85 km^2^ in July and 322.78 km^2^ in February, respectively. There was also an increasing trend from February to July and a decreasing trend from July to February.

### 3.3. Spatial Distribution

**Figure 5:**
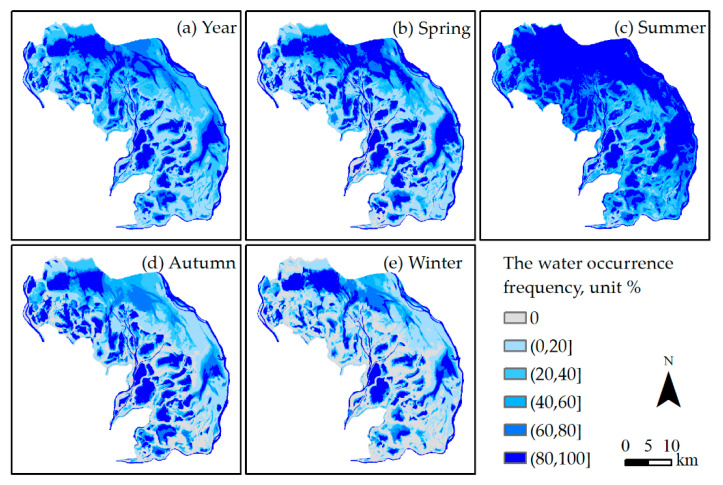
Water occurrence frequency during the study period from 24 May 2015 to 2 June 2020 for the (**a**) year; (**b**) spring; (**c**) summer; (**d**) autumn; and (**e**) winter.

As shown in Figure 5 the water occurrence frequency is the probability that water exists in a specific period. We defined the water body with a water occurrence frequency of more than 80% as permanent water in a specific period. Figure 5a illustrated that the area of permanent water reached 272.55 km^2^, which accounted for 25.74% of the total area of the study region during the study period. More than half of the area was covered by permanent water throughout the summer, which was distributed primarily in the north and east parts of the study region, as shown in Figure 5c. During winter, the permanent water area was the smallest compared with the other seasons and accounted for 17.91% of the total area of the study region.

The permanent water presents a spatial form of isolated sub-lakes or rivers, as shown in Figure 5, which can be connected during the flood seasons. Statistics indicate that the average number of sub-lakes with area of more than 0.1 km^2^ was 129, with a standard deviation of 43, during the study period. In addition, the average number of sub-lakes with area of more than 1 km^2^ was 37, with an average area of 54 km^2^.

### 3.4. Influence of Climate

**Figure 6:**
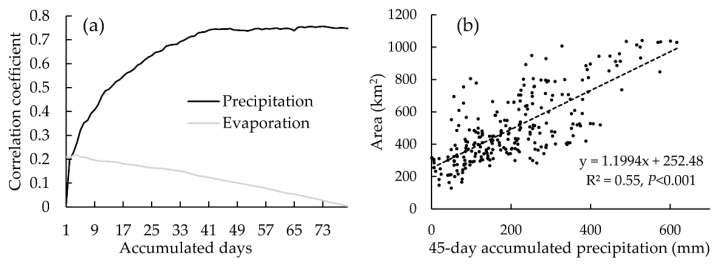
(**a**) Correlation coefficient between the accumulated precipitation and evaporation with different days; and (**b**) scatter plot of the 45-day accumulated precipitation and the surface water area.

There were 255 pairs of data for surface water area, evaporation and precipitation. The correlation analysis results showed that correlation coefficient between the surface water area and the accumulated evaporation within 4-day was 0.22 (*p* < 0.01), which was the highest observed value, as shown in Figure 6a. The correlation coefficient between the accumulated precipitation and surface water area generally increased along with the number of cumulative days and reached a peak near the 45th day, as shown Figure 6a. Therefore, we believe that the accumulated precipitation within the 45-day period correlates well with the surface water area with a correlation coefficient of 0.75 (*p* < 0.001), as shown in Figure 6b. The regression equation is shown as follows:

(4) y=1.1994×x+252.48

where *y* is the surface water area (km^2^) and *x* is the 45-day accumulated precipitation (mm).

**Figure 7:**
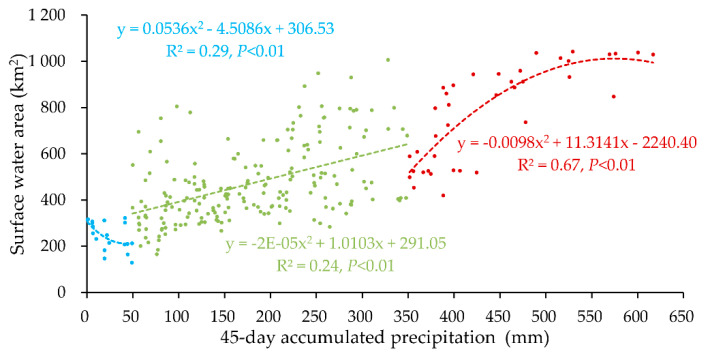
The prediction models for surface water area based on the 45-day accumulated precipitation.

To better predict the surface water area, we divided these 255 pairs of data into 3 groups based on 45-day accumulated precipitation to build three piecewise prediction models. The three prediction models shown in Figure 7 are quadratic equations in one variable between surface water area, which is the dependent variable, and 45-day accumulated precipitation, which is the independent variable. The expressions were shown in Equation (5). When the independent variable is less than 50 mm (drought), between 50 mm to 350 mm (moist), and more than 350 mm (flooding), the first model (blue), the second model (green), and the third model (red)was used to predict surface water area, respectively. Each model was significant (*p* < 0.01).

(5) $$y=\left\{ \begin{array}{l} if\;x<50\mathrm{mm},\;0.0536x^{2}-4.5086x+306.53\\ if\;50\leq x<350\mathrm{mm},\;-2E-05x^{2}+1.0103x+291.05\\ if\;x\geq 350\mathrm{mm},\;-0.0098x^{2}+11.3141x-2240.40 \end{array}\right.$$

where *y* is the surface water area (km^2^) and *x* is the 45-day accumulated precipitation (mm).

**Figure 8:**
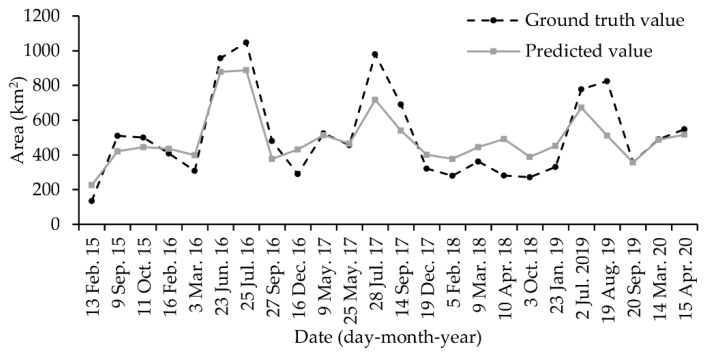
Validation for the prediction model. Ground truth values were derived from Landsat-8 images unaffected by clouds.

To validate the reliability of the prediction model, we collected all free-cloud Landsat-8 images during January 2015 to July 2020 to extract surface water area, which were regarded as ground truth value to compare with corresponding prediction value. The result is depicted in Figure 8. According to 24 pairs of ground truth value and prediction value, the mean absolute error (MAE) was 92.62 km^2^, which accounted for 18.33% of the average of ground truth value, and the root mean square error (RMSE) was 119.32 km^2^.

Validation results demonstrated that the model was significantly underestimated in July and August. For different seasons, the MAE in the spring, summer, autumn, and winter was 62.75, 148.28, 87.03 and 86.69 km^2^, respectively. The RMSE in the spring, summer, autumn, and winter was 93.14, 183.31, 98.91 and 95.09 km^2^, respectively.

## 4. Discussion

**Figure 9:**
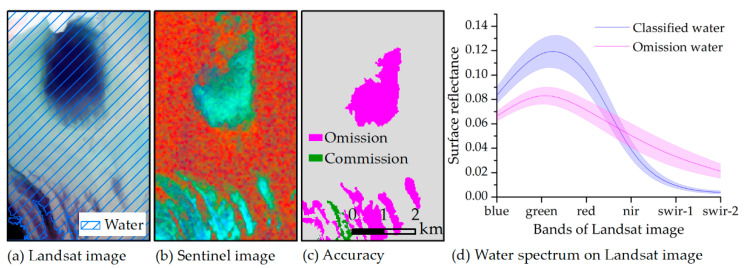
Image characteristics for omission water from the Landsat-8 and Sentinel-1 imagery obtained on 9 September 2015. (**a**) Landsat true-color image with R: red band, G: green band, and B: blue band. The area covered by diagonal lines is identified as water. (**b**) Sentinel-1 false-color composition image with R: SWI image, G: VH image, and B: VV image, where the red region is water. (**c**) Classification accuracy of the SWI-based water extraction model (SWIM) compared with the Landsat classification results. (**d**) The spectrum for classified water and omission water on Landsat-8 image, where the lines represent the mean value and the shade represents the standard deviation.

The SAR and optical satellite imagery have an excellent recognition ability to identify water bodies, as demonstrated by previous research [53,54]. However, there are still some uncertainties in the identification of water bodies in complex environments with the confluence of water transparency, plankton, submerged plants, emerged plants, and suspended solids. For example, there is a black patch in Figure 9a and the Landsat classification results indicate that is water. However, its color is quite different from that of the surrounding water. Conversely, the black patch is barely recognized as water by the Sentinel-1 imagery because it fails to have a distinctive water feature, as shown in Figure 9b. A similar phenomenon also exists in the south for Figure 9c and other areas of the study region. Despite these findings, the mechanism behind the phenomenon requires more relevant investigations in the future for clarity. Compared with the spectral characteristics of classified water by Sentinel-1 imagery, the reflectance of omission water on the green band of Landsat-8 imagery is lower while that on the SWIR band is higher. As a result, the MNDWI value of omission water ranges from 0.3 to 0.6 but is more than 0.7 for most classified water. Most omission water exists in the boundaries of lakes and some narrow rivers. Previous research has also reported similar omission errors [55].

**Figure 10:**
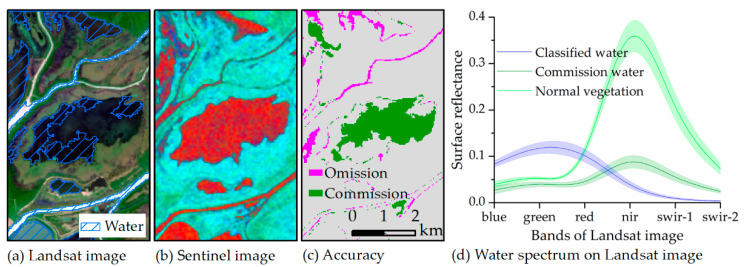
Image characteristics for commission water for the Landsat-8 and Sentinel-1 imagery obtained on 9 September 2015. (**a**) Landsat true-color image with R: red band, G: green band, and B: blue band. The area covered by diagonal lines is water as identified by Landsat-8 imagery. (**b**) Sentinel false-color composition image with R: SWI image, G: VH image, and B: VV image, where the red region is water. (**c**) the classification accuracy of the SWIM compared to the Landsat classification result, and (**d**) the spectrum for classified water, commission water, and normal vegetation from the Landsat-8 imagery. The lines represent the mean, and the shade represents the standard deviation.

We also studied the spectral characteristics of commission water, which was identified as water by Sentinel-1 but as non-water by Landsat-8 imagery and found that its spectrum was similar to that of vegetation. As there are many aquatic plants in the commission water area, the reflectance is lower in the green band of the Landsat-8 imagery and is comparatively higher in the NIR and SWIR bands compared with classified water. Thus, Landsat-8 imagery cannot recognize these areas as water. In fact, the surface of these areas was water and very smooth. Hence, they have similar image features as classified water for the Sentinel-1 imagery, as shown in Figure 10b.

Precipitation is among the important factors that drive the dynamics of surface water areas for southwest Poyang Lake, and these conclusions are consistent with previous studies [10,56]. However, Figure 3 illustrates that the correlation between the surface water area and the accumulated precipitation is very low in some periods. Feng et al. [10] noted that there is no significant correlation between the surface water area of the entire Poyang Lake region and local precipitation during the summer months of July to September because the Yangtze River backflows into Poyang Lake, which could lead to a rapid increase in surface water area during summers. According to Figure 3, this phenomenon, i.e., Yangtze River backflows into Poyang Lake, also occurs in other months, e.g., October and March, because of the Three Gorges Dam’s flood discharge [57,58,59]. Poyang Lake is connected to the Yangtze River and is one of the important flood diversion areas of the Yangtze River. Under normal circumstances, the water of Poyang Lake flows out through the Yangtze River. When the Yangtze River is flooded, the water level of the Yangtze River is higher than the water level of Poyang Lake, then a backflow event occurs, resulting in the rapid increase in water surface of Poyang Lake. Therefore, the prediction model proposed in the study suffers from an underestimation issue when Yangtze River backflows into Poyang Lake. For example, the ground truth value is 824.19 km^2^ on 19 August 2019, but the predicted value is 511.05 km^2^. We believe that introducing a correction parameter according to the amount of water backflow into Poyang Lake from the Yangtze River could significantly improve the performance of prediction model in the future.

Conversely, the accumulated precipitation reached 600 mm from 14 April 2018 to 26 May 2018, but the water area remained the same. The rapid increase in precipitation was caused by heavy rains on April 14 and 15 (196 mm). Before this, there was a drought period of up to 5 months. Therefore, after a long period of continuous drought, the contribution of one heavy precipitation on the lake expansion is minor. A similar phenomenon occurred in January and February 2020. In this case, the prediction model has an overestimation issue.

Theoretically, the surface water area and evaporation should show a negative correlation; however, our experiments show a positive correlation between the two variables. The correlation coefficient between the surface water area and the 4-day accumulated evaporation was 0.22 (*p* < 0.01), which was the highest observed value. This may be because the surface water area and evaporation have similar variation trends within a year (increasing from winter to summer and decreasing from summer to winter). Compared with the total revenue and expenditure of the water volume of southwest Poyang Lake, the impact of evaporation on the water volume is considered insignificant.

Wetlands are among Earth’s most productive systems [60]. Our study area includes a part of the Poyang Lake National Nature Reserve [60] and the entire Nanjishan Wetland National Nature Reserve [61], which is an important wetland resource in China. Poyang Lake is a valuable natural resource and has regional ecological functions, such as climate regulation, flood control, and wildlife habitats [56,62,63,64]. However, in recent decades, Poyang Lake is under considerable threats from intensive human activities and dramatic climate change. For example, urban sewage inputs have led to the eutrophication of the lake water, and sand dredging has resulted in highly turbid lake water, and flood and drought events are becoming increasingly frequent [21,65,66]. As shown in Figure 3, there were two serious drought events that occurred in early 2017 and late 2019, which lasted for several months. It is of great scientific significance to further study the impact of continuous drought events on the ecosystem of Poyang Lake based on the map of surface water.

## 5. Conclusions

The SWI has great potential in surface water identification. Our study showed that the average overall accuracy in water classification attained 91.92% in southwest Poyang Lake using the SWI-based water extraction model. However, there still exist some uncertainties in the identification of water bodies based on the Sentinel-1 and Landsat-8 imagery, especially in complex environments such as those affected by plankton and submerged plants. Besides, narrow rivers and water boundaries are generally where classification errors often occur.

The surface water area of southwest Poyang Lake is characterized by dramatic fluctuations at intra-month and inter-month scales. For example, within a 6-day interval, the maximum changed area was 233.42 km^2^ from 474.70 km^2^ on 20 March 2017 up to 708.12 km^2^ on 26 March 2017. For most of the year, southwest Poyang Lake is divided into numerous isolated sub-lakes that are connected only during flood periods.

The prediction model, which was built based on 45-day accumulated precipitation, performs well in predicting southwest Poyang Lake surface water area according to the validation results using Landsat-derived ground truth data. Nevertheless, the model has an underestimation issue when Yangtze River backflows into Poyang Lake. This is because the contribution of the Yangtze River backflow to the increase of lake area is much greater than that of precipitation.

## Appendix A

The function of following codes is to implement Sentinel-1 images processing on Google Earth Engine cloud computing platform for this study. The codes runnable link as follows: https://code.earthengine.google.com/da1544aef0d6ca84df9d388611549e73.

var addSWI = function(image)

  {return image.addBands(image.expression(

    '0.1747*VV+0.0082*VH*VV+0.0023*VV*VV-0.0015*VH*VH+0.1904',

    { 'VH': image.select('VH_1'),

    'VV': image.select('VV_1') } ).rename('swi')); };

var kernel = ee.Kernel.square({radius: 1});//window 3*3

var medianFilter = function(image){return image.addBands(image.focal_median({kernel: kernel, iterations: 1}))};

var geometry = ee.Geometry.Rectangle([115.9,28.6,116.5,29.3,]);

Map.setCenter(116.2,29.0,9);

var m = 0;

var num = 1; //Number of processed Sentinel-1 images

var start_date = ee.Date('2020-1-10'); // Start date for processing Sentinel-1 images

for (var i = 0; i < num; i++) {

  var end_dat = start_date.advance(1,'day');

  var s1 = ee.ImageCollection('COPERNICUS/S1_GRD')

    .filterBounds(geometry)

    .filterDate(start_date,end_dat)

    .map(medianFilter)

    .map(addSWI);

  var nn = s1.size().getInfo();

  if(nn === 0) {print('Missing data:',start_date);}

  if (nn > 0){

    var s1_image = s1.mean();

    var s1_swi = s1_image.select('swi').toFloat().clip(geometry);

    var swi_class = s1_swi.gt(0.2).unmask(0).int8().clip(table).rename('water');

    var s1_list = s1.toList(nn);

    var id = (ee.Image(s1_list.get(0))).id().getInfo().toString()

    var id_infor = 'swi_gt02_'

    for (var j = 17; j <25;j++){

      var id_infor = id_infor+id[j] }

    var id_infor = id_infor+'_'+id[0]+id[1]+id[2]

    var m = m+1;

    var vectors = swi_class.reduceToVectors({

      geometry: geometry,

      crs: swi_class.projection(),

      scale: 30,

      geometryType: 'polygon',

      eightConnected: false,

      labelProperty: 'swi', });

    Export.table.toDrive( {

      collection:vectors,

      description:'shp_'+id_infor,

      fileNamePrefix:'shp_'+id_infor,

      fileFormat:'SHP',

      folder: 'swi_vector_gt02' });

  }//end if

  var start_date = start_date.advance(6,'day');

}//end for
